# Prevalence of Self-Reported Work-Related Lower Back Pain and Its Associated Factors in Ethiopia: A Systematic Review and Meta-Analysis

**DOI:** 10.1155/2021/6633271

**Published:** 2021-09-23

**Authors:** Mihretu Jegnie, Mekbeb Afework

**Affiliations:** ^1^Department of Biomedical Sciences, College of Health Sciences, Debre Tabor University, Debre Tabor, Ethiopia; ^2^Department of Anatomy, College of Health Sciences, Addis Ababa University, Addis Ababa, Ethiopia

## Abstract

**Introduction:**

Low back pain is the commonest musculoskeletal disorder affecting every socioeconomic group of the world's population. The lifetime risk of developing low back pain is about 60%–80%. The pooled prevalence and associated factors of low back pain have not yet been determined in Ethiopia. Thus, this study was aimed at assessing the overall prevalence of low back pain and its associated factors in Ethiopia.

**Methods:**

A systematic search of PubMed, Scopus, Science Direct, and Google Scholar for observational studies reporting data on the prevalence and associated factors of low back pain was conducted. Relevant data were extracted with a standardized data extraction excel form. Stata 14 was employed for the meta-analysis. Heterogeneity was assessed by Cochran's *Q* test and *I*^2^ values of a forest plot. Publication bias was checked using a funnel plot and Egger's test. A random-effects model was used in the analysis.

**Result:**

A total of thirty-two studies were included for the systematic review. Twenty-four and sixteen studies were used to pool the overall low back pain prevalence and associated factors, respectively. The overall pooled annual prevalence of low back pain in Ethiopia was estimated to be 54.05% (95% CI: 48.14–59.96). Age, sex, body mass index, work experience, working hours, lack of safety training, awkward working posture, work shift, prolonged standing, lifting heavy objects, sleeping disturbance, history of back trauma, previous medical history of musculoskeletal disorder, and lack of adequate rest interval at work were significantly associated with low back pain.

**Conclusion:**

The current systematic review and meta-analysis revealed a higher prevalence of lower back pain in Ethiopia. Most of the low back pain epidemiological studies conducted in Ethiopia focused on specific occupational settings, making pooling of data and comparison with other countries challenging. Thus, further general population studies are recommended.

## 1. Introduction

Lower back pain (LBP) is the most common musculoskeletal disorder (MSD) affecting the adult population, with a lifetime prevalence of up to 84% [1, 2]. The pain is felt by patients from the anatomical area of the back below the twelfth rib and above the inferior gluteal fold [3]. The symptoms can arise from many potential anatomic sources, such as nerve roots, muscles, fasciae, bones, joints, intervertebral discs (IVDs), and organs within the abdominal cavity [2, 4, 5]. The lumbar vertebrae are the main anatomical framework of the lower back. The lumbar vertebrae are interconnected by joint capsules, ligaments, tendons, and muscles. It has extensive innervation and vascular supply. The vertebral column is designed to be strong since it has to protect the spinal cord and spinal nerve roots. At the same time, it is highly flexible, providing for mobility in many different planes [2].

On average, 95% of low back disorders are nonspecific or strain/sprain. The pain may arise from any of the spinal structures: intervertebral disc, facets, ligaments, vertebrae, tendons, and muscles [6, 7]. Conventionally, the origins of LBP are grouped under four categories: discogenic/neurological, muscular/ligamentous, structural, and other disorders [8].

Work-related LBP is any back pain considered clinically to have been probably caused, at least in part, or aggravated by job [9]. It causes considerable absence from work and loss in productivity, resulting in financial burdens to employers, employees, and healthcare system [10]. It was identified as one of the top three occupational health problems to be targeted by the World Health Organization (WHO) [11].

Previous investigations have explored that LBP is the cause for an estimate of 818,000 and 21.8 million disability-adjusted life years (DALYs) in 2000 [10] and 2010 [12], respectively. Approximately, 60% to 80% of people will suffer from LBP at some point in their lifetime [12].

Globally, low back and neck pain were ranked as the 12th, 8th, and 4th leading cause of DALYs in 1990, 2005, and 2015, respectively. From 1990 to 2015, low back and neck pain DALYs increased by 59.5%. In 2015, low back and neck pain were the leading cause of disability in most countries (the second leading cause of DALYs in high-income countries next to ischemic heart disease) [13, 14].

The global 12-month prevalence of LBP was estimated to be 38% from a systematic review of the general population studies published between 1980 and 2009. The mean point prevalence was 18.3%. Its lifetime prevalence was also estimated to be 38.9% [15]. However, in two earlier global LBP prevalence systematic reviews, one using studies conducted from 1954 to 1993 [16] and the other using studies conducted from 1966 to 1998 [17], pooling of data was not possible as a result of methodological variability between studies. The annual, point, and lifetime LBP prevalence in the general population ranged from 22% to 65%, 12 to 33%, and 11% to 84%, respectively, according to the systematic review conducted using studies all over the world from 1966 to 1998 [17].

A systematic review of 27 studies conducted in Africa revealed that the mean LBP point prevalence among adolescents was 12%, while among adults, it was 32%. The average one-year prevalence of LBP among adolescents and adults was 33% and 50%, respectively. The average lifetime prevalence of LBP among adolescents was 36% and among adults was 62%. The most common population on which research had been conducted was “workers” (48%) [18].

Another recent systematic review in Africa revealed the pooled lifetime, annual, and point prevalence of LBP to be 47% (95% CI: 37, 58), 57% (95% CI: 51, 63), and 39% (95% CI: 30, 47), respectively [19].

LBP is the leading common MSD. It accounted for 81.3% [20] and 60.2% [21] in India and Nigeria, respectively, from total MSDs. Its prevalence varies across the job type. Particularly, the prevalence rate is increased among agricultural workers, carpenters, welders, drivers, truck and tractor operators, nurses, cleaners, and domestic assistants [9]. For instance, the 12-month prevalence of LBP was 76.6% among operating room nurses and 75.1% among X-ray technologists in Netherlands [22]. A recent meta-analysis showed that LBP is the most prevalent work-related MSD (62%) out of the nine musculoskeletal body regions among perioperative nurses [23].

Low back pain is a multifactorial problem having many possible etiologies. Determining risk factors for LBP is therefore a difficult task. A multitude of determinants of LBP and sciatica includes sociodemographic and individual factors, physical factors, and psychosocial factors [24].

Age, gender, physical activity, anthropometry, medical history, and socioeconomic status were identified as determinant factors of LBP by several studies. Evidence has suggested that middle age, female gender, and increased BMI are risk factors for LBP [15, 18, 24–36]. Cigarette smoking was also associated with LBP in some studies [7, 32, 37].

Many factors causing LBP can be associated with job. Globally, about 37% of LBP can be attributed to work [10]. Several studies have identified physical factors, such as frequent bending, twisting, overstretching, vibration, heavy weight lifting, heavy weight pulling and pushing, prolonged standing, prolonged sitting, and heavy physical work load as predictors of LBP [20, 24, 38–44].

Factors such as job dissatisfaction, low social support from colleagues or supervisors, job stress/high workload, feeling little pleasure, sleeping disturbance, and depression were also implicated to be associated with LBP in different studies [38, 45, 46].

The overall prevalence and risk factors of work-related LBP in Ethiopia have not yet been investigated. Thus, the main aim of this systematic review and meta-analysis was to estimate the pooled prevalence and identify the factors associated with work-related LBP in Ethiopia. Moreover, this study has also assessed the methodological quality of available studies. The results of this meta-analysis will help policy-makers and other concerned bodies to plan and implement strategies to prevent the impacts of LBP. It will also help researchers to improve future research quality on LBP in particular and musculoskeletal pain in general as the methodological quality of previous studies was critically appraised. The review question is as follows: what are the overall prevalence and associated risk factors of work-related LBP in Ethiopia?

## 2. Methods

### 2.1. Search Strategy

Observational studies reporting the prevalence and associated factors of LBP in Ethiopia were gathered by using the following search approaches:

- Search for published journal articles using international scientific databases including PubMed, Google Scholar, Science Direct, and Scopus
- Search for unpublished MSc/PhD thesis reports using Google, Google Scholar, and local university websites

**Figure 1:**
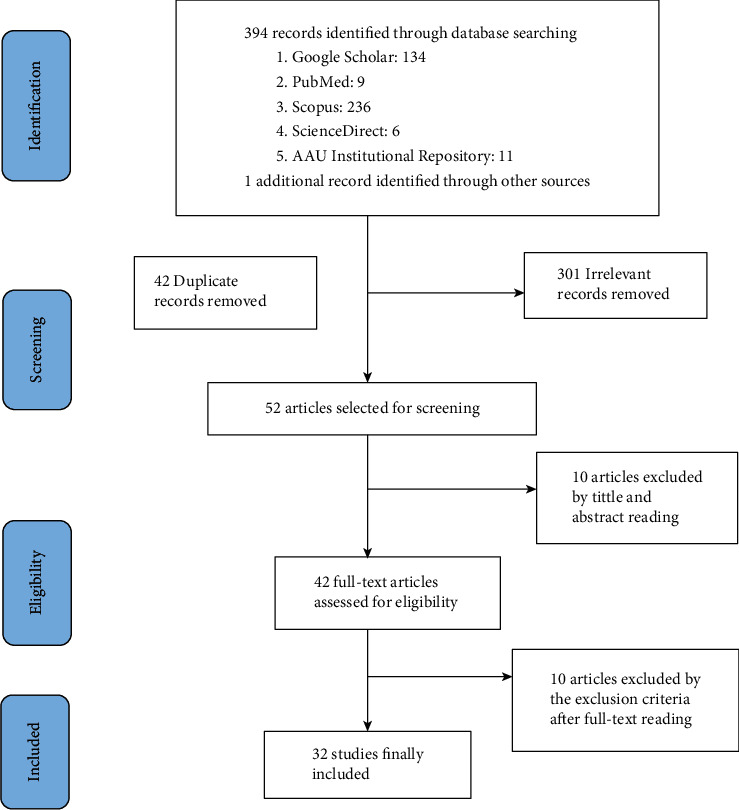
PRISMA flowchart describing the selection of studies for the systematic review and meta-analysis of low back pain prevalence and associated factors in Ethiopia.

Study articles were retrieved by using search terms like (“prevalence” OR “magnitude” OR “epidemiology” OR “burden”) AND (“associated factors” OR “risk factors” OR “factors”) AND (“low^*∗*^ back pain” OR “back pain” OR “musculoskeletal disease” OR “musculoskeletal disorder OR musculoskeletal pain”) AND “Ethiopia.” All fields and MeSH terms were used to search PubMed. MeSH terms were not used to search the rest of the databases. The first search was done until the end of November 2019 and repeated on July 30, 2020. No date range restrictions were employed. Following the initial search, the articles that were included were also reference searched to exhaustively retrieve the articles. Preferred Reporting Items for Systematic Reviews and Meta-Analyses (PRISMA) guideline was strictly followed as shown in Figure 1. The full search strategy is available upon request from the corresponding author.

### 2.2. Criteria for Considering Studies for This Review

#### 2.2.1. Inclusion Criteria

Observational studies (cross-sectional, cohort, and case-control) having relevant extractable data regarding the prevalence and associated factors conducted in Ethiopia at the community or facility level were included in the review. Both published and unpublished articles in the English language were included.

#### 2.2.2. Exclusion Criteria

Research articles that did not report the outcome of interest after reviewing were excluded. Studies conducted on specific age or sex categories were also excluded from the meta-analysis part because it creates more heterogeneity between studies.

### 2.3. Data Extraction

Important data including author, publication year, number of study participants, study design, study area, sampling procedure, prevalence of LBP, and risk factors with their odds ratio were extracted using a consistent Microsoft Excel format.

Variables mentioned in two or more included studies were selected to be included in this systematic review and meta-analysis. Moreover, the variables should be significantly associated with LBP in at least one included study.

### 2.4. Methodological Quality Assessment of Studies

The methodological quality of the included studies was assessed using the “Newcastle-Ottawa Quality Assessment tool adapted for cross-sectional studies” [47], which was previously used by Leboeuf-Yde and Lauritzen, 1995 [16], Walker, 2000 [17], Louw, 2007 [18], and Morris, 2018 [19] for a similar purpose (see Additional file 1). The tool contains three parts: representation of the target population, data quality, and case definition of the LBP. The tool has ten items, each having equal weight, and the total score was 100. “The Joanna Briggs Institute (JBI) Critical Appraisal Checklist for Case Control Studies, 2017” [48] was used to appraise one case-control study (see Additional file 1). Two of the authors (MJ and MA) independently evaluated the methodological quality of the studies. Differences in opinion between the reviewers were discussed until consensus was reached.

### 2.5. Statistical Analysis

Review data were extracted using Microsoft Excel format and exported to Stata/SE 14 for analysis. Forest plot was done to check heterogeneity between the studies. In the forest plot, *χ*^2^ test, *I*^2^ test, and *P* values were checked for heterogeneity. A value of zero indicates true homogeneity, while values of 25%, 50%, and 75% indicate low, moderate, and high heterogeneity, respectively [49]. Due to an anticipated heterogeneity, a random-effect Der Simonian and Laird's model [50] was applied to estimate the pooled prevalence of LBP.

Possible publication bias was also evaluated objectively by using Egger's correlation and Begg's regression intercept tests at a 5% significant level [51]. Furthermore, to reduce the random discrepancies between the point estimates of the primary study, subgroup analysis was carried out based on occupation of the study participants and the region where the studies were conducted. A univariate metaregression model was also carried out to identify the possible sources of heterogeneity by considering study characteristics, such as publication year and sample size. A metaregression analysis was performed to assess the different factors associated with LBP.

### 2.6. Definition of Terms

#### 2.6.1. LBP

LBP is the experience of pain, ache, or discomfort, felt below the costal margin and above the inferior gluteal folds, with or without leg pain for at least one-day duration [3].

#### 2.6.2. Lifetime Prevalence

Lifetime prevalence is the experience of LBP at any point in the individual's lifetime.

#### 2.6.3. Annual Prevalence

Annual prevalence is the experience of LBP at any point in the past 12 months.

#### 2.6.4. Point Prevalence

Point prevalence is the experience of LBP at the time of the study's data collection.

#### 2.6.5. Body Mass Index (BMI)

Weight in kilograms is divided by the square of the height in meters (kg/m^2^) [52]:

1. Underweight: BMI <18.50
2. Normal: BMI of 18.50–24.99
3. Overweight = BMI b/*n* 25.00–29.99
4. Obese = BMI ≥30.00

#### 2.6.6. Satisfaction

An employee will be considered satisfied with a job when his/her sum of generic job satisfaction scale score is 32 or above [53].

#### 2.6.7. Physical Exercise

It refers to performing any kind of physical exercise at least two times per week for 30 minutes [54].

#### 2.6.8. Job Stress

Job stress is a score measured using the workplace stress scale as yes (16 to 40) and no (lower than or equal to 15) [55].

#### 2.6.9. Cigarette Smoking

Cigarette smoking is the practice of smoking at least one stick of cigarette per day [56].

#### 2.6.10. Alcohol Drinking

Alcohol drinking is the consumption of any kind of alcohol at least two times a week.

#### 2.6.11. Lifting Heavy Objects

It refers to lifting, carrying, pulling, or pushing heavy loads weighing 25 kilograms and above every day or every other day.

#### 2.6.12. Health and Safety Training

It refers to any kind of training in a one-year period through any kind of media about health and safety rules related to one's own occupation.

## 3. Results

### 3.1. Study Identification

A search of the important databases yielded a total of 394 articles. One unpublished MSc thesis was also found. After removal of 42 duplicate and 301 unrelated articles, 52 studies were selected for in-depth screening. After title and abstract screening, 42 full-text articles were assessed for eligibility. 10 articles were again excluded by the exclusion criteria after full-text reading. 32 articles were finally included for the review and meta-analysis (Figure 1). The search strategy and results obtained using PubMed are shown in Additional file 2. A list of the excluded studies and the reasons for their exclusion is available from the corresponding author.

### 3.2. Characteristics of Included Studies

A total of 32 observational studies with a sample size of 13,859 respondents were included in this review. The characteristics of the included studies are shown in Tables 1 and 2. The articles range in date from 2009 to 2020 with the mean year of publication being 2017. 75% of the included studies were published or conducted during and after the year 2017. The majority of the included studies [31] were cross-sectional by design. Most of the studies were conducted in Addis Ababa (11 studies) and Oromia (10 studies). The other eight studies were conducted in two regions: 6 in Amhara and 2 in Tigray.

In those studies that reported data about gender distribution, 49.64% of the respondents were females. However, data on gender distribution were missing in three of the included studies, namely, Lette et al. [75], Lamina et al. [73], and Wanamo et al. [66]. The mean age of respondents ranges from 11.5 (Delele et al. [88]) to 49 (Jeon and Jeon [87]) years. 62.5% of the studies included respondents aged between 25 and 35 years.

Sample size varied from 100 [73] to 814 [68] (mean = 433.09 ± 162.93); response rate varied from 82.3% [67] to 100% [57, 66, 69–71, 74, 76] (mean = 95 ± 5.23). Three studies (Jeon and Jeon [87], Yitayeh et al. [72], and Abir et al. [86]) did not report the response rates. In the case-control study, Deksisa et al. [82], the response rate is not applicable. In 22 of the studies, an interviewer-administered standardized questionnaire was used as a data collection tool, while in the rest 9 studies, self-administered questionnaire was employed. In one study, Abir et al. [86], a combination of questionnaire, interview, and document review was employed. The questionnaire employed by most of the included studies was adapted from the Standardized Nordic Questionnaire for the Analysis of Musculoskeletal Symptoms (SNQAMS) [3]. 30 of the studies reported data about the prevalence of LBP, whereas 18 studies recorded data about the factors associated with the development of LBP.

### 3.3. Quality Assessment Results of Included Studies

The mean methodological quality score of the 32 included studies was 84.51%. 12.5% of the studies scored 100%. Criteria item 2 is not applicable to 7 of the studies because their response rate is 100%. Considering the mean methodological score, the authors determined arbitrarily the threshold for acceptable study quality to be 70%. Two studies, Abir et al., [86] and Jeon and Jeon, [87], were excluded from further analysis as they scored below 70% (Table 3).

### 3.4. Definition of Low Back Pain

Out of the thirty-two studies included, only eleven specified the minimum duration of the episode to be counted as a case of LBP. Seven of them specified the minimum duration as “2–3 days,” while four of them specified the minimum duration as “at least one day.” Twenty-five studies stated the anatomic location of the pain in their case definition. Fourteen mentioned the area as “the lower back, lumbar, or buttock area” and the rest eleven identified it as “the area of the back below the 12th rib (costal margin) and above the inferior gluteal fold.” Six studies did not provide the case definitions of LBP.

### 3.5. Prevalence of Low Back Pain

The 12-month prevalence of LBP ranges from 3.21% in office workers to 82.93% in nurses. There were about 6,753 cases of self-reported LBP out of the 13,265 study participants of the 30 studies that reported magnitude. The mean annual prevalence of LBP was 50.69% ± 17.75. All primary studies retrieved focused on a specific occupation. The magnitude and related factors associated with LBP were well studied in the nursing profession (9 studies) and industry workers (6 studies). There was no LBP prevalence study found conducted on the general population in Ethiopia. All were done using a certain specific occupation.

The mean prevalence of LBP in the studies that defined the anatomical area as the “posterior aspect of the body from the lower margins of the twelfth ribs to the lower gluteal folds” (50.39%) was comparable with that in studies that did not specify the anatomic location (50.06%).

All studies, except Deksisa et al. [82] and Zungu [81], reported an annual (12 months) prevalence of LBP. Only five studies [58, 59, 80, 83, 84] reported seven days prevalence. The mean seven-day prevalence (37.52%) was lower than the mean annual prevalence (50.69%). Point prevalence was reported only in two studies: Belay et al. [65] (45.32%) and Mijena et al. [85] (36.39%). Lifetime prevalence was reported in only one study (Abebe et al. [60] (50.6%)).

### 3.6. Pooled Prevalence of Low Back Pain

Not all studies included in the systematic review were pooled in the meta-analysis to keep homogeneity. Pooling of studies was evaluated not only by methodological quality but also by their similarity in the study population (general or working), age, sex, and definition of LBP. Considering this evaluation criterion, in addition to the studies excluded by poor methodological quality, four more studies were excluded objectively from the main meta-analysis. These were Delele [88] (because it used only children and youngsters less than the age of 18 years); Tefera et al. [68] (because it used only young adolescents in the age of 14–17 years); Yosef et al. [62] (because it used only female respondents); and Henok [74] (because it used only male respondents).

Twenty-four studies were therefore finally pooled to estimate the prevalence of LBP in Ethiopia. The studies selected for the pooling of LBP prevalence were those studies that reported similar recall periods of LBP prevalence; studies that used both genders; studies that did not use a specific age population; studies that mentioned a similar anatomic location of pain. All included studies in the systematic review were done on a certain working population. Although the difference in the type of work the study subjects were engaged in was noted, the pooling of the data was decided by the authors as work-related LBP. Therefore, the data pooled in this meta-analysis included the adult working population of both genders.

**Figure 2:**
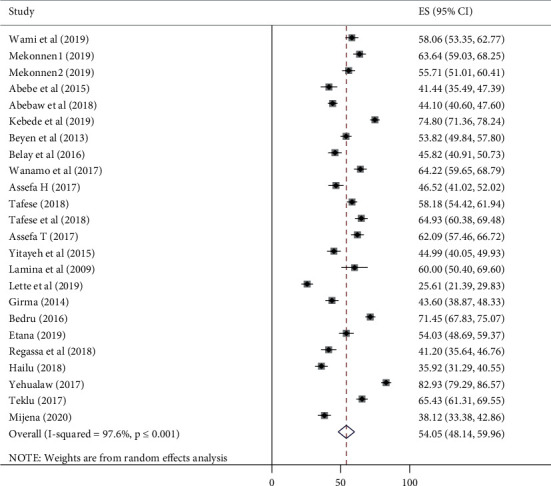
Forest plot of the pooled annual prevalence of low back pain in Ethiopia.

The overall pooled 12-month prevalence of self-reported work-related LBP from 24 Ethiopian studies yielded 54.05% (95% CI: 48.14–59.96) (Figure 2). Only five studies provided data about seven-day LBP prevalence and the pooled prevalence was estimated to be 37.48% (95% CI: 22.63, 52.33). The pooled point LBP prevalence (40.82%) was estimated from only two studies. There was no sufficient number of studies to pool the lifetime prevalence of LBP. The *I*^2^ statistic revealed considerable heterogeneity between the studies (*I*^2^ = 97.6, *P* ≤ 0.001). Hence, a random-effects model was used to estimate the pooled prevalence of LBP in Ethiopia.

### 3.7. Subgroup Analysis

**Figure 3:**
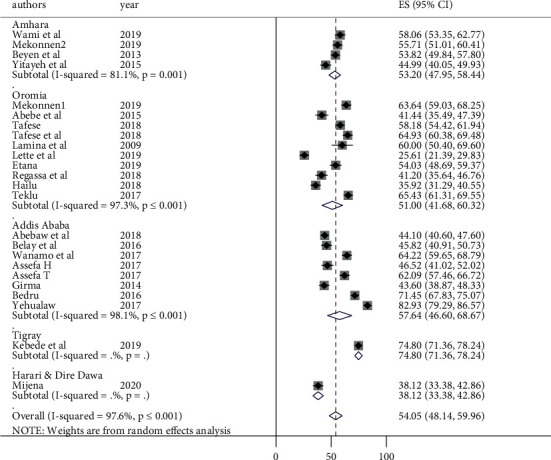
Forest plot of subgroup analysis by region and prevalence of low back pain in Ethiopia.

**Figure 4:**
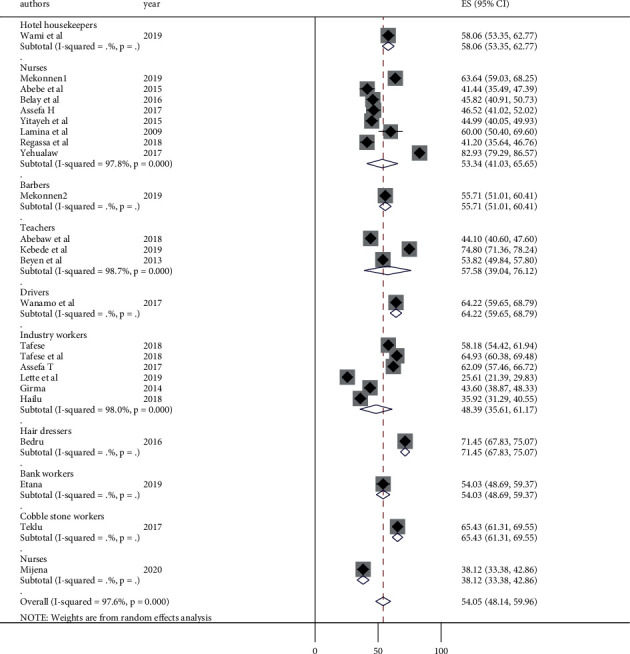
Forest plot of subgroup analysis by occupation and prevalence of low back pain in Ethiopia.

**Figure 5:**
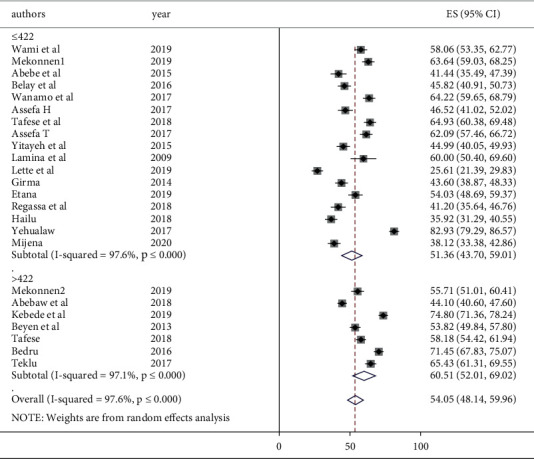
Forest plot of subgroup analysis by sample size and prevalence of low back pain in Ethiopia.

**Figure 6:**
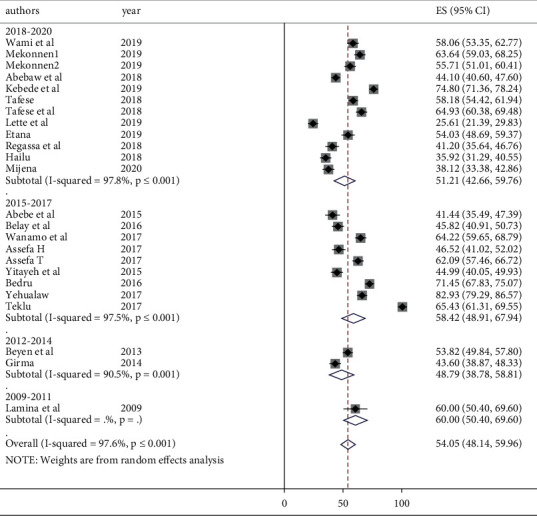
Forest plot of subgroup analysis by publication year and prevalence of low back pain in Ethiopia.

To identify the possible sources of heterogeneity among the included studies, subgroup analysis was performed using occupation, region, sample size, and publication year to estimate the overall prevalence of LBP. Administrative regions of Ethiopia and occupations mentioned by the primary studies were used for grouping. The minimum adequate sample size for a cross-sectional study (422) was used as a cut-off value to group by sample size. In order to group by publication year, three consecutive years were grouped together by judgment. Accordingly, the highest LBP burden was observed among the studies conducted in Tigray region (74.8%) and the lowest was observed to be in Harari and Dire Dawa (38.12%) (Figure 3). Subgroup analysis by occupation revealed that the lowest LBP was observed among industry workers with a prevalence of 48.39% (95% CI: 35.61, 61.17) (Figure 4). The highest prevalence was observed among hair dressers (71%). The subgroup analysis by sample size also showed a significant difference between studies conducted with a sample size >422 (60.51%) and ≤422 (51.36%) (Figure 5). The prevalence of LBP was found to be 60%, 48.79%, 58.42%, and 51.21% in studies published between 2009 and 2011, 20012 and 2014, 2015 and 2017, and 2018 and 2020, respectively (Figure 6).

### 3.8. Sensitivity Analysis

There were six studies excluded from the main meta-analysis with reason. The annual LBP prevalence in these six studies ranged between 3.21% and 65%. Had these six studies been included to estimate the pooled LBP prevalence in the meta-analysis, it would have been 50.68%. The pooled annual prevalence of LBP in all the 30 studies identified reporting magnitude (including the six studies already excluded) was found to be 50.68% (95% CI: (41.12, 60.24)). The pooled annual prevalence of LBP using the 24 methodologically sound studies was 54.05% (95% CI: 48.14–59.96). Thus, limiting the analysis of studies based on methodological quality score therefore significantly increased the annual LBP prevalence from 50.68% to 54.05%.

### 3.9. Publication Bias

**Figure 7:**
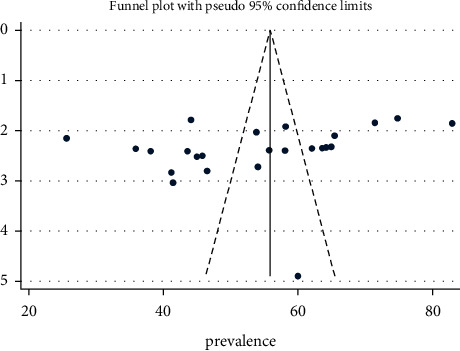
Funnel plot to test publication bias of the included studies.

Both the subjective visualization of the funnel plot (Figure 7) and the result of Eger's test (*P*=0.076) (Table 4) indicated the absence of publication bias in this review.

### 3.10. Factors Associated with Low Back Pain

Various risk factors assessed by the original articles as listed in Tables 5 and 6 were selected for analysis in this study. Using Stata/SE14 software, the association of the aforementioned risk factors with the development of LBP was analyzed. Accordingly, 14 of the selected factors were found to be significantly associated with LBP. These were age, sex, BMI, work experience, working hours, lack of safety training, repetitive awkward working posture (bending, twisting, and overstretching), work shift, prolonged standing, lifting heavy objects, sleeping disturbance, history of back trauma, previous medical history of MSD, and lack of adequate rest interval at work.

**Figure 8:**
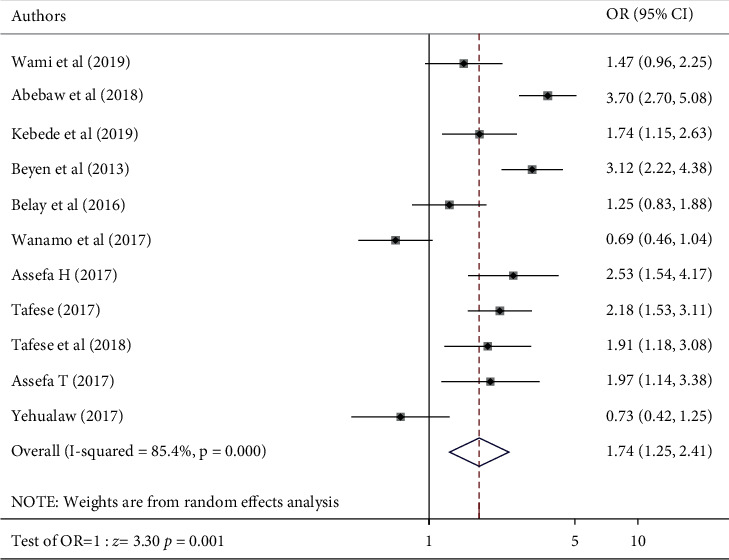
Forest plot of included studies investigating the association between age and low back pain in Ethiopia.

**Figure 9:**
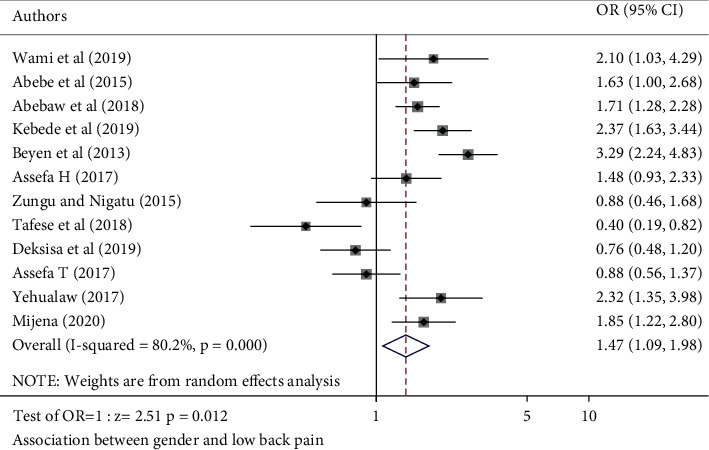
Forest plot of included studies investigating the association between gender and low back pain in Ethiopia.

Respondents who were 30 and above years of age were 1.74 times more likely to develop LBP than those who were below 30 (POR: 1.74, 95% CI (1.25, 2.41)) (Figure 8). The odds of developing LBP were 1.47 times more likely among females than males (Figure 9). Respondents whose BMI was greater than or equal to 25 kg/m^2^ had 1.62 times odds of developing LBP compared to those whose BMI was below 25.

Study participants who had five or more years of work experience were 2.26 times more likely to have LBP than those of workers with work experience of less than five years. Those who work for 8 or more hours per day had 2.69 times odds of developing LBP compared to those who work for less than 8 hours. The likely hood of developing LBP among respondents who lacked health and safety training increased by 2.16 times compared to those who took the training. Respondents whose job requires assuming awkward working postures such as repetitive bending, twisting, and overstretching had increased odds of having LBP by 197% (POR: 2.97) compared to those whose task does not require. The other work-related factor significantly associated with LBP was heavy weight lifting. Respondents who were involved in heavy weight lifting activities were 1.49 times more likely to have LBP than those who were not involved.

Workers who had both shifts (day and night) were 1.61 times more likely to develop LBP than those who worked only in the day shift (POR = 1.61; 95% CI: 1.11, 2.32). Workers who had a history of back trauma were 3.46 times more likely to develop LBP than those who did not have trauma. Those who had a previous medical history of MSD were 5.06 times more likely to have LBP than those who did not have.

## 4. Discussion

The 12-month prevalence of LBP in the current systematic review ranges from 3.21% in office workers to 82.93% in nurses. However, limiting the reports only to methodologically sound studies changed the range from 25.61% to 82.93%. As stated in other similar systematic reviews [15–19, 89], it is very challenging to compare and pool the prevalence of LBP between populations as there are considerable methodological variabilities across studies. All previous attempts to pool the prevalence of LBP were not successful because of methodologic heterogeneity and poor qualities of studies. Some of the various methodological differences include variability in sample size; differences in LBP case definition, particularly prevalence period, minimum duration of pain, and anatomic location of the pain; using nonstandard tools for measurement of outcomes; and heterogeneity in the study population such as age and sex. However, the improvement in methodologic homogeneity, especially the relative similarity in data collection tools and LBP case definitions seen in the studies included in our review, justified the pooling of LBP prevalence.

On the other hand, comparison of the current systematic review and meta-analysis with other previous systematic reviews is difficult and should be interpreted cautiously. This is because the current review involved the working population while previous reviews involved the general population [15, 17], the elderly [90, 91], adolescents [92], and children [93].

The current one-year pooled prevalence of LBP in Ethiopia (54.05% (95% CI: 48.14–59.96)) was comparable with a mean estimate of Nigeria (55.39%) [89]. The 12-month prevalence of LBP in Nigeria ranged from 32.5% to 73.53% [89], which is different from the current finding (25.61% to 82.93%). All reviewed studies were occupation-based and did not depict a true general population prevalence of LBP in both the present review and the case of Nigeria. Another systematic review conducted in Africa reported a 12-month prevalence of LBP ranging from 14% to 72% [18]. The current meta-analysis overall estimate lies in this range.

The pooled annual prevalence of LBP in Ethiopia (54.05%) was more or less comparable with a study in Botswana (55.7%) [39] and the recent systematic review conducted using African studies published after 2006 (57%) [19]. The lowest prevalence observed in the African studies systematic review (51%) was higher than the current lowest prevalence (25.6%).

A systematic review of LBP using studies conducted between 1966 and 1998 by Walker [17] revealed a point prevalence ranging from 12% to 33%; one-year prevalence ranging from 22% to 65%; and lifetime prevalence ranging from 11% to 84%. A higher annual LBP prevalence range (25.61% to 82.93%) was observed in the current systematic review and meta-analysis since the studies included were done on the vulnerable population (working population), whereas Walker [17] included only studies conducted on the general population, excluding the working population.

In a systematic review of general population studies conducted from 1980 to 2009, the global 12-month prevalence was estimated to be 38%, which is much lower than the current meta-analysis (54.05%). This discrepancy is likely to be due to the variation in the study population (general versus working population). The mean point prevalence from the same systematic review was 18.3%, which is again lower than our current point prevalence estimate (40.86%).

Another possible cause for the high pooled prevalence demonstrated in our current meta-analysis could be the age range of the respondents in the included studies apart from the inclusion of only studies conducted on a working population. As noted above in the Results section, in the majority of the studies, the respondents' mean age ranges from 25 to 35 years. Therefore, the inclusion of the middle-aged adult population in the review might have contributed to the higher prevalence of LBP as also reported in previous studies [15, 25, 94, 95].

Our subgroup analysis showed the annual prevalence of LBP among nurses to be 53.4%, which is in agreement with a study done in India (53.4%) [96], but lower than the studies in Iran (74.3%) [97], Nepal (75.7%) [98], and Uganda (58.7%) [99]. It was also lower than the systematic review done in Africa among nurses (64.07%) [100]. Such differences can be attributed to the general work environment variation between health service settings across countries, particularly related to the variation in the number of nursing staff and workload. The other possible reason for the higher prevalence among nurses in other countries could be the higher proportion of female-to-male ratio in the nursing profession compared to Ethiopia. The annual pooled prevalence of LBP among industry workers in our subgroup analysis was 48.39% (95% CI: 35.61, 65.19). It was lower than the studies conducted using a similar industrial population in Iran (57.1%) [101], Kosovo (61.6%) [102], and Nigeria (59.5%) [103]. A comparison with previous studies is shown in Table 7.

The results of the present systematic review and meta-analysis found out that several physical, sociodemographic, and individual factors are associated with LBP.

The current meta-analysis of risk factors showed age to significantly affect the chance of developing LBP, which is in line with previous systematic reviews such as Louw [18], Hoy [25], Hoy [15], Manchikanti [24], and Jordaan [26]. According to a study done in India [32] among textile industry workers, age ≥35 years was found to have 9 times more risk than age <35 years (AOR = 9.45; 95% Cl: 5.24,17.01). The increased occurrence of LBP as age advances can be explained by increased disc degeneration and decreased elasticity of ligaments. The prevalence of disc degeneration was 34% in 20–39, 59% in 40–59, and 93% in 60–80 years old persons in MRI study [104]. Lumbar spine stability diminishes following lumbar disc degeneration. An unstable lumbar spine then imposes a higher biomechanical demand on the ligaments, capsules, muscles, and facets [9].

Our current analysis of risk factors revealed that LBP was more prevalent in the female gender, which is consistent with other systematic reviews such as Louw [18], Jordaan [26], and Wang [27]. Osteoporosis, menstruation, pregnancy, and giving birth could contribute to the increased prevalence of LBP in females compared to males [15].

The other individual factor found to be associated with LBP was BMI. This finding was consistent with other studies in the United States [36], Israel [34], Norway [35], and India [32]. Among textile industry workers in India [32], obese subjects were highly affected by LBP (AOR = 9.14; 95% CI: 4.95, 16.87). In a meta-analysis of 33 studies by Shiri [33], obesity was associated with increased prevalence of LBP (POR: 1.33; 95% CI: 1.14, 1.54) in cross-sectional studies and (POR = 1.53; 95% CI: 1.22, 1.92) in cohort studies. Although several literature studies mentioned the association of BMI with LBP, the exact mechanism of how increased BMI or body height causes LBP remains unclear [34].

Work experience of more than five years, working more than eight hours per day, and working in both shifts (day and night) were associated with an increased risk of LBP compared to their counterparts. Similar reports from the United States [105] supported our finding of increased working hours spent on repeated activities and back pain. The association of work shift and LBP was also demonstrated in other reviews and studies [40, 41]. The increased occurrence of LBP in those who work in both shifts might be due to disturbance of the normal circadian rhythm and increased workload during the nighttime.

The other significantly associated factor identified was health and safety training. Workers who took ergonomics training (occupational health and safety training) were less likely to report LBP than those who did not. A similar finding was observed in Hong Kong [106]. It is obvious that workers who are aware of ergonomic precautions are likely to implement preventive measures and will reduce their exposure to MSDs including LBP.

Similar to the reports of Bongers et al. [38] and Malikraj [20], our study demonstrated that awkward working posture (frequent bending, twisting, and overstitching) is a major risk factor for LBP. A similar finding was also reported from a study in Nigeria [42], Saudi Arabia [43], and Malaysia [44]. Frequent bending and twisting were reported to be the most frequent causes of back injuries in England [107]. Lateral bending and twisting while lifting heavy objects were found to be harmful [108, 109]. Bad body postures during work such as twisting, leaning, bending, kneeling, overstretching, and squatting are believed to increase spinal stress and accelerate degenerative changes in the disc and other structures [110, 111].

Previous findings indicated that types of jobs that require static work postures, such as prolonged standing and sitting, increase the risk of developing LBP [24, 112], which is consistent with our current findings. Prolonged standing causes excessive strain on the lumbar spine and other anatomical structures responsible for the development of MSDs, including LBP.

Workers involved in heavy weight lifting had an eight times higher incidence of low back injuries than sedentary work [113]. Another prospective cohort study also identified heavy weight lifting as an important physical risk factor [38]. Heavy physical strain, frequent lifting, vibration, and postural stress are likely to result in disc degeneration and LBP [24].

Although the relationship can be two-sided, sleeping disturbance was significantly associated with LBP in the current meta-analysis. Another recent meta-analysis [114] revealed a similar association but with relatively lower odds (1.52) than the present study (2.07).

Having a history of back trauma as well as a previous medical history of MSD was associated with increased risk of LBP in the present work with higher odds than all other risk factors. A history of work-related low back injury was positively associated with LBP in a study done in Canada [115] (AOR = 3.66; 95% CI: 2.48, 5.42). A history of musculoskeletal disease was also associated with an increased risk of LBP (OR = 7.1) in a study conducted in Vietnam [116].

The current systematic review and meta-analysis did not show a statistically significant association between physical exercise and LBP, which is consistent with other previous systematic reviews [117, 118]. However, in other systematic reviews, conflicting evidence has been reported between leisure-time physical activity and LBP. For instance, Hosam et al. in their systematic review and meta-analysis of cross-sectional studies found a significant reduction in LBP among individuals who perform physical exercise [119]. Similarly, Shiri and Falah Hassani [28] reported a decreased risk of chronic LBP among individuals who engaged in medium and high-level leisure-time physical activities in a meta-analysis of cohort studies. On the other hand, a systematic review of observational studies reported an increased risk of LBP among individuals who perform strenuous physical activity [120].

None of the psychosocial factors in the analysis of risk factors in this meta-analysis showed a significant association with LBP, although some of the included studies have reported associations. As opposed to our findings, studies elsewhere showed low job satisfaction, high workload, and decreased social support from colleagues or supervisors to be associated with LBP [38, 45, 46]. This difference could be attributed to the variation in the social work environment and workload between Ethiopia and other countries. Cigarette smoking, alcohol drinking, night shift, and employment status did not show a significant association with LBP in this meta-analysis of risk factors.

## 5. Limitations

Hotel housekeepers, hair dressers, drivers, barbers, bank workers, and cobble stone workers were each represented in the systematic review and meta-analysis by only one study. Most of the significant work-related factors of LBP in these studies were not included in the analysis because the factors should be found in at least two studies.

Extracting data on risk factors from the original studies was a challenging task as there is no homogeneity in constructing and measuring the factors. As a result, some studies were omitted from the analysis of risk factors, and this could introduce bias.

It should be noted that causal relationships cannot be established from the results of the factor analysis since most of the studies included in the meta-analysis were cross-sectional by design.

## 6. Conclusion

This systematic review and meta-analysis revealed a high burden of LBP in the working segment of the Ethiopian population. A high burden of LBP among teachers, nurses, industry workers, drivers, hair dressers, hotel housekeepers, cobble stone workers, and bank workers was noted. Ergonomic, individual, and sociodemographic factors associated with LBP have been identified and their strength of association has also been measured in the present work. The current systematic review and meta-analysis indicated that implementing appropriate interventional ergonomics programs in the workplace and providing training to the workers could help reduce LBP incidence.

The authors recommend conducting a more comprehensive study involving the general population at the community level. Moreover, future studies shall have uniformity in their methodology, especially in terms of data collection tools and case definition so that further pooling and comparison will be possible. Only one study has been found conducted among agricultural field workers. Further research may be needed to investigate the situation of LBP in this significant segment of the Ethiopian population. Further large-scale prospective studies are needed to determine if the associations of LBP with the factors identified in this meta-analysis are causal.

## Figures and Tables

**Table 1:** Characteristics of the included studies, part one: publication year, setting, population, region, and design of the study.

Authors	Year	Study setting and population	Region	Design
Wami et al. [57]	2019	Hotel housekeepers	Amhara	C/S
Mekonnen [58]	2019	Public hospital nurses	Oromia	C/S
Mekonnen [59]	2019	Barbers	Amhara	C/S
Abebe et al. [60]	2015	Hospital staff	Oromia	C/S
Abebaw et al. [61]	2018	Governmental primary schools	AA	C/S
Yosef et al. [62]	2019	Long-distance truck drivers, Modjo dry port	AA	C/S
Kebede et al. [63]	2019	Primary school teachers	Tigray	C/S
Beyen et al. [64]	2013	Primary and secondary school teachers	Amhara	C/S
Belay et al. [65]	2016	Public hospitals	AA	C/S
Wanamo et al. [66]	2017	Taxi drivers	AA	C/S
Assefa [67]	2017	Hospital nurses	AA	C/S
Tefera et al. [68]	2019	Traditional young weavers	SNNPR and AA	C/S
Tafese [69]	2018	Ammunition engineering industry workers	Oromia	C/S
Tafese et al. [70]	2018	Garment industry workers	Oromia	C/S
Assefa [71]	2017	Metal and engineering industry welders	AA	C/S
Yitayeh et al. [72]	2015	Governmental health institution nurses	Amhara	C/S
Lamina et al. [73]	2009	Hospital nurses	Oromia	C/S
Henok and Bekele [74]	2017	Backloading women	SNNPR	C/S
Lette et al. [75]	2019	Building construction workers	Oromia	C/S
Girma [76]	2014	Selected garment workers	AA	C/S
Bedru [77]	2016	Female beauty salon hair dressers	AA	C/S
Etana [78]	2019	Banks workers	Oromia	C/S
Regassa et al. [79]	2018	Public hospital nurses	Oromia	C/S
Hailu [80]	2018	Bishoftu automotive industry workers	Oromia	C/S
Zungu and Nigatu [81]	2015	Ethiopian airlines aircraft technicians	AA	C/S
Deksisa et al. [82]	2019	Low back pain patients in health institutions	Oromia	C/C
Yehualaw [83]	2017	Public and private hospitals nurses	AA	C/S
Teklu [84]	2017	Cobble stone workers	AA	C/S
Mijena et al. [85]	2020	Public hospital nurses	Harari and DD	C/S
Abir et al. [86]	2017	Woreda offices workers	Amhara	C/S
Jeon and Jeon [87]	2017	Rural community farmers	Tigray	C/S
Delele et al. [88]	2018	Elementary school children	Amhara	C/S

AA: Addis Ababa; DD: Dire Dawa; SNNPR: Southern Nations, Nationalities, and Peoples' Region; C/S: cross-sectional; C/C: case control.

**Table 2:** Characteristics of the included studies, part two: sampling technique, data collection tool, sample size, response rate, mean age, and low back pain cases.

Authors	Sampling technique	Data collection tool	Sample	Response rate (%)	Mean age and SD	Gender		LBP cases
						F	M	
Wami et al. [57]	Systematic	IAQ^*∗*^	422	100	26.71 ± 4.9	388	34	245
Mekonnen1 [58]	Systematic	IAQ^*∗*^	418	99	31.39 ± 7.01	233	185	266
Mekonnen2 [59]	Systematic	IAQ^*∗*^	429	98.8	26.38 ± 4.78	56	373	239
Abebe et al. [60]	Simple	IAQ	263	90.7	33.5 ± 6.7	126	137	109
Abebaw et al. [61]	Cluster and simple	IAQ^*∗*^	771	93.2	33 ± 10.63	378	393	340
Yosef et al. [62]	Systematic	IAQ^*∗*^	400	94.8	37.7 ± 9.13	0	400	260
Kebede et al. [63]	Simple	SAQ^*∗*^	611	93	40 ± 9.38	331	280	457
Beyen et al. [64]	Stratified	SAQ	602	90.9	38 ± 11.02	191	411	324
Belay et al. [65]	Simple	SAQ	395	91.9	30.6 ± 8.4	285	110	181
Wanamo et al. [66]	Systematic	IAQ^*∗*^	422	100	35.28 ± 10.06	NR	271	
Assefa [67]	Convenience	SAQ^*∗*^	316	82.3	31.6 ± 8.4.	193	123	147
Tefera et al. [68]	Multistage	IAQ^*∗*^	814	99	14–17 years	221	593	398
Tafese [69]	Simple	IAQ^*∗*^	660	100	30.1 ± 8.1	211	449	384
Tafese et al. [70]	NR	IAQ^*∗*^	422	100	26.9 ± 7.2	370	52	274
Assefa [71]	Simple	IAQ^*∗*^	422	100	26 (MA)	112	310	262
Yitayeh et al. [72]	Census	IAQ^*∗*^	389	NR	30 ± 5.8	209	180	175
Lamina et al. [73]	Simple	SAQ	100	83	NR	NR	60	
Henok and Bekele [74]	Convenience	IAQ^*∗*^	422	100	>15	422	0	255
Lette et al. [75]	Simple	SAQ	410	97.2	31.32 ± 7.54	NR	105	
Girma [76]	Stratified and simple	IAQ^*∗*^	422	100	29 (MA)	355	67	184
Bedru [77]	Multistage	IAQ^*∗*^	599	98.3	25 (MA)	472	127	428
Etana [78]	Simple	IAQ^*∗*^	335	98	31 ± 5.27	80	255	181
Regassa et al. [79]	Systematic	SAQ	301	90.4	NR	142	159	124
Hailu [80]	Simple	IAQ^*∗*^	412	97.6	28.6 ± 5.7	155	257	148
Zungu and Nigatu [81]	Randomly	SAQ	294	93	31	43	251	NR
Deksisa et al. [82]	Proportional	IAQ	300	NA	30 (MA)	132	168	NA
Yehualaw [83]	Census	IAQ^*∗*^	410	91.5	28.67 ± 5.46	306	104	340
Teklu [84]	Simple	IAQ^*∗*^	512	95	NR	102	410	335
Mijena et al. [85]	Simple	SAQ	404	96	30.4 ± 8.733	225	179	154
Abir et al. [86]	Simple and purposive	IAQ and DR	343	NR	NR	212	131	11
Jeon and Jeon [87]	Cluster	IAQ	116	NR	49	43	73	46
Delele et al. [88]	Multistage	IAQ^*∗*^	723	85.6	11.5 ± 2.7	423	297	50

IAQ: interviewer-administered questionnaire; F: female; SAQ: self-administered questionnaire; DR: document review; NR: not reported; M: male; MA: median age; SD: standard deviation; NA: not applicable.^*∗*^Standardized Nordic questionnaire for the analysis of musculoskeletal symptoms; .

**Table 3:** Methodological quality assessment results of the included studies.

Authors	Evaluation criteria (see Additional file 1)										%
	1	2	3	4	5	6	7	8	9	10	
Wami et al. [57]	√	NA	√	√	√	√	√	√	√	√	100
Mekonnen1 [58]	√	X	√	√	√	√	√	X	√	√	80
Mekonnen2 [59]	√	X	√	√	√	√	√	√	√	√	90
Abebe et al. [60]	√	X	√	√	√	√	√	√	√	√	90
Abebaw et al. [61]	√	X	√	√	√	√	√	√	X	√	80
Yosef et al. [62]	√	X	√	√	√	√	√	√	√	√	90
Kebede et al. [63]	√	X	√	√	√	√	√	√	√	√	90
Beyen et al. [64]	√	X	√	√	√	√	√	√	√	√	90
Belay et al. [65]	√	X	√	√	√	√	√	√	√	√	90
Wanamo et al. [66]	√	NA	√	√	√	√	√	X	√	√	88.88
Assefa [67]	X	X	X	√	√	√	√	√	√	√	70
Tefera et al. [68]	√	X	√	√	√	√	√	X	√	√	90
Tafese [69]	√	NA	√	√	√	√	√	X	√	√	88.88
Tafese et al. [70]	X	NA	√	√	√	√	√	√	√	√	88.88
Assefa [71]	√	NA	√	√	√	√	√	√	√	√	100
Yitayeh et al. [72]	√	X	X	√	√	√	√	√	X	√	70
Lamina et al. [73]	√	X	√	√	√	√	√	√	√	√	90
Henok and Bekele [74]	X	NA	√	√	√	√	√	√	X	√	77.77
Lette et al. [75]	√	X	√	√	√	√	√	X	√	√	80
Girma [76]	√	NA	√	√	√	√	√	√	√	√	100
Bedru [77]	√	X	√	√	√	√	√	√	√	√	90
Etana [78]	√	X	√	√	√	√	√	√	√	√	90
Regassa et al. [79]	√	√	√	√	√	√	√	√	√	√	100
Hailu [80]	√	X	√	√	√	√	√	√	√	√	90
Zungu and Nigatu [81]	√	X	√	√	√	√	√	√	√	√	90
Deksisa et al. [82]	√	√	√	√	√	X	X	√	√	√	80
Yehualaw [83]	√	X	√	√	√	√	√	√	√	√	90
Teklu [84]	√	X	√	√	√	√	√	√	X	√	80
Mijena et al. [85]	√	X	√	√	√	√	√	X	√	√	80
Abir et al. [86]	√	X	X	X	X	X	X	√	√	X	30
Jeon and Jeon [87]	X	X	X	√	√	√	√	X	X	√	50
Delele et al. [88]	√	X	√	√	√	√	√	√	√	√	90

√: criteria fulfilled; X: criteria were not fulfilled; NA: not applicable.

**Table 4:** Egger's test to test publication bias of the included studies.

Std. eff.	Coef.	Std. err.	*t*	*P*>|t|	(95% conf. interval)
Slope	83.34189	15.55954	5.42	≤0.001	51.3252 116.6104
Bias	−12.7094	6.83008	−1.86	0.076	−26.27056 1.456976

**Table 5:** Factors associated with low back pain.

Factors	POR (CI)	*I* ^2^	Chi-square	*P* value
Age, years				
<30 (reference)	1			
≥30	1.74 (1.25, 2.41)	85.4	68.28	**0.001** ^*∗*^

Sex				
Male	1			
Female	1.47 (1.09, 1.98)	80.2	55.56	**0.012** ^*∗*^

BMI, kg/m^2^				
<25	1			
≥25	1.62 (1.26, 2.08)	24.7	7.97	**≤0.001** ^*∗*^

Work experience, years				
<5	1			
≥5	2.26 (1.66, 3.08)	83.9	74.42	**≤0.001** ^*∗*^

Alcohol drinking				
No	1			
Yes	1.34 (0.69, 2.63)	77.1	8.73	0.387

Working hours, hours				
≤8				
>8	2.69 (1.58, 4.60)	76.8	12.9	**≤0.001** ^*∗*^

Took safety training				
Yes	1			
No	2.16 (1.66, 2.80)	60.2	15.06	**≤0.001** ^*∗*^

Physical activity/exercise				
Yes	1			
No	1.45 (0.91, 2.31)	90.3	103.06	0.118

Job stress				
No	1			
Yes	1.23 (0.62, 2.41)	93.0	71.57	0.556

Awkward working posture				
No	1			
Yes	2.97 (1.85, 4.77)	75.4	16.27	**≤0.001** ^*∗*^

Work shift				
Day shift only	1			
Both shifts	1.61 (1.11, 2.32)	62.8	8.06	**0.011** ^*∗*^

Felt little pleasure by doing things				
No	1			
Yes	2.01 (0.95, 4.24)	79.7	4.93	0.069

**Table 6:** Factors associated with low back pain (continued).

Factors	POR (CI)	*I* ^2^	Chi-square	*P* value
Night shift				
No	1			
Yes	2.88 (0.81, 10.21)	66.5	2.98	0.101

Prolonged standing				
No	1			
Yes	2.53 (1.41, 4.57)	83.6	12.20	**0.002** ^*∗*^

Prolonged sitting				
No	1			
Yes	1.19 (0.74, 1.92)	41.6	1.71	0.480

Cigarette smoking				
No	1			
Yes	1.67 (0.79, 3.49)	81.9	37.66	0.177

Lifting heavy objects				
No	1			
Yes	1.49 (1.13, 1.96)	47.7	11.48	**0.005** ^*∗*^

Sleeping disturbance				
No	1			
Yes	2.07 (1.23, 3.47)	85	20.04	**0.006** ^*∗*^

History of back trauma				
No	1			
Yes	3.46 (2.11, 5.67)	65.8	8.77	**≤0.001** ^*∗*^

Job satisfaction				
Yes	1			
No	1.06 (0.47, 2.37)	96.3	134.09	0.892

Previous medical history of MSD				
No	1			
Yes	5.06 (2.86, 8.96)	35.4	1.55	**≤0.001** ^*∗*^

Employment status				
Permanent	1			
Temporary	0.82 (0.40, 1.66)	84.5	12.88	0.580

Adequate rest interval at work				
Yes	1			
No	1.86 (1.37, 2.53)	54.8	8.88	**≤0.001** ^*∗*^

^*∗*^Value shows a significant association at a significance level of 0.05; POR: pooled odds ratio; CI: confidence interval; *I*^2^: heterogeneity test score.

**Table 7:** Comparison of low back pain prevalence of the current study with others.

Author		Walker [17]	Hoy [15]	Louw [18]		Morris [19]	Bello [89]	Current (based on the 24 included studies)	
Country		Global	Global	Africa		Africa	Nigeria	Ethiopia	
				Ado	Adult			Pooled	Mean
Point prevalence in %	M	—	18.3	12	32	39	33.28	40.82	40.86
	L	12	6.6	10	16	30	14.7	36.39	36.39
	H	33	30	14	59	47	59.7s	45.32	45.32
Annual prevalence in %	M	—	38	33	50	57	55.39	54.05	50.69
	L	22	18.1	14	—	51	32.5	25.61	25.61
	H	65	43.5	—	72	63	73.53	82.93	82.93
Lifetime prevalence in %	M	—	38.9	36	62	47	51.75	—	—
	L	11	14.6	28	—	37	45.5	—	—
	H	84	63.2	—	74	58	58	—	—
Seven-day prevalence in %	M	—	—	—	—	—	—	37.48	37.52
	L	—	—	—	—	—	—	15.29	15.29
	H	—	—	—	—	—	—	54.15	54.15

Ado: adolescents; M: mean; L: lowest; H: highest; —: data not reported.

## Data Availability

The datasets used and/or analyzed during the current study are available from the corresponding author upon reasonable request.

## Abbreviations

AOR: Adjusted odds ratio
BMI: Body mass index
IVD: Intervertebral discs
CI: Confidence interval
LBP: Low back pain
MRI: Magnetic resonance imaging
MSD: Musculoskeletal disorder
OR: Odds ratio
POR: Pooled odds ratio
SNQAMS: Standardized Nordic questionnaire for the analysis of musculoskeletal symptoms
WHO: World Health Organization.

## Supplementary Materials

Supplementary Materials The methodological quality of the included studies was assessed using “Newcastle-Ottawa Quality Assessment Tool Adapted for Cross-Sectional Studies” shown in Additional file 1. The search strategy and results obtained using PubMed are shown in Additional file 2.

## References

[1] Balagué F., Mannion A. F., Pellisé F., Cedraschi C. (2012). Non-specific low back pain. *Lancet*.

[2] Allegri M., Montella S., Salici F. (2016). Mechanisms of low back pain: a guide for diagnosis and therapy. *F1000Research*.

[3] Kuorinka I., Jonsson B., Kilbom A. (1987). Standardised Nordic questionnaires for the analysis of musculoskeletal symptoms. *Applied Ergonomics*.

[4] Smart K. M., Blake C., Staines A., Thacker M., Doody C. (2012). Mechanisms-based classifications of musculoskeletal pain: Part 1 of 3: symptoms and signs of central sensitisation in patients with low back (±leg) pain. *Manual Therapy*.

[5] Garland E. L. (2012). Pain processing in the human nervous system: a selective review of nociceptive and biobehavioral pathways. *Primary Care: Clinics in Office Practice*.

[6] Frank J. W., Kerr M. S., Brooker A.-S. (1996). Disability resulting from occupational low back pain. *Spine*.

[7] Putz-Anderson V., Bernard B. P., Burt S. E. (1997). *Musculoskeletal Disorders and Workplace Factors*.

[8] Khalil T. M. (1993). *Ergonomics in Back Pain: A Guide to Prevention and Rehabilitation*.

[9] De Beeck R. O., Hermans V. (2000). *Work-Related Low Back Disorders*.

[10] Punnett L., Prüss-Ütün A., Nelson D. I. (2005). Estimating the global burden of low back pain attributable to combined occupational exposures. *American Journal of Industrial Medicine*.

[11] Choi B. C. K., Tennassee L. M., Eijkemans G. J. M. (2001). Developing regional workplace health and hazard surveillance in the Americas. *Revista Panamericana de Salud Públic*.

[12] Hoy D., March L., Brooks P. (2014). The global burden of low back pain: estimates from the Global Burden of Disease 2010 study. *Annals of the Rheumatic Diseases*.

[13] Hurwitz E. L., Randhawa K., Yu H., Côté P., Haldeman S. (2018). The Global Spine Care Initiative: a summary of the global burden of low back and neck pain studies. *European Spine Journal*.

[14] GBD DALYs and HALE Collaborators (2015). Global, regional, and national disability‐adjusted life‐years (DALYs) for 315 diseases and injuries and healthy life expectancy (HALE), 1990–2015: a systematic analysis for the Global Burden of Disease Study 2015. *Lancet*.

[15] Hoy D., Bain C., Williams G. (2012). A systematic review of the global prevalence of low back pain. *Arthritis and Rheumatism*.

[16] Leboeuf-Yde C., Lauritsen J. M. (1995). The prevalence of low back pain in the literature A structured review of 26 nordic studies from 1954 to 1993. *Spine*.

[17] Walker B. F. (2000). The prevalence of low back pain: a systematic review of the literature from 1966 to 1998. *Journal of Spinal Disorders*.

[18] Louw Q. A., Morris L. D., Grimmer-Somers K. (2007). The prevalence of low back pain in Africa: a systematic review. *BMC Musculoskeletal Disorders*.

[19] Morris L. D., Daniels K. J., Ganguli B., Louw Q. A. (2018). An update on the prevalence of low back pain in Africa: a systematic review and meta-analyses. *BMC Musculoskeletal Disorders*.

[20] Malikraj S., Senthil Kumar T., Ganguly A. K. (2011). Ergonomic intervention on musculoskeletal problems among welders. *International Journal of Advances in Engineering and Technology*.

[21] Chiang H. C., Ko Y. C., Chen S. S., Yu H. S., Wu T. N., Chang P. Y. (1993). Prevalence of shoulder and upper-limb disorders among workers in the fish-processing industry. *Scandinavian Journal of Work, Environment and Health*.

[22] Bos E., Krol B., van der Star L., Groothoff J. (2007). Risk factors and musculoskeletal complaints in non-specialized nurses, IC nurses, operation room nurses, and X-ray technologists. *International Archives of Occupational and Environmental Health*.

[23] Clari M., Godono A., Garzaro G. (2021). Prevalence of musculoskeletal disorders among perioperative nurses: a systematic review and META-analysis. *BMC Musculoskeletal Disorders*.

[24] Manchikanti L. (2000). Epidemiology of low back pain. *Pain Physician*.

[25] Hoy D., Brooks P., Blyth F., Buchbinder R. (2010). The epidemiology of low back pain. *Best Practice and Research Clinical Rheumatology*.

[26] Jordaan R., Kruger M., Stewart A. V., Becker P. J. (2005). The association between low back pain, gender and age in adolescents. *South African Journal of Physiotherapy*.

[27] Wáng Y. X. J., Wáng J.-Q., Káplár Z. (2016). Increased low back pain prevalence in females than in males after menopause age: evidences based on synthetic literature review. *Quantitative Imaging in Medicine and Surgery*.

[28] Shiri R., Falah-Hassani K. (2017). Does leisure time physical activity protect against low back pain? Systematic review and meta-analysis of 36 prospective cohort studies. *British Journal of Sports Medicine*.

[29] Shiri R., Karppinen J., Leino-Arjas P., Solovieva S., Viikari-Juntura E. (2010). The association between obesity and low back pain: a meta-analysis. *American Journal of Epidemiology*.

[30] Holmström E. B. (1992). Musculoskeletal disorders in construction workers related to physical, psychosocial, and individual factors. *Acta Orthopaedica Scandinavica*.

[31] Latza U., Karmaus W., Stürmer T., Steiner M., Neth A., Rehder U. (2000). Cohort study of occupational risk factors of low back pain in construction workers. *Occupational and Environmental Medicine*.

[32] Tiwari R. R., Pathak M. C., Zodpey S. P. (2003). Low back pain among textile workers. *Indian Journal of Occupational and Environmental Medicine*.

[33] Chowdhury D., Sarkar S., Rashid M. H., Rahaman A., Sarkar S. K., Roy R. (2014). Influence of body mass index on low back pain. *Mymensingh Medical Journal: MMJ*.

[34] Hershkovich O., Friedlander A., Gordon B. (2013). Associations of body mass index and body height with low back pain in 829,791 adolescents. *American Journal of Epidemiology*.

[35] Heuch I., Hagen K., Heuch I., Nygaard Ø., Zwart J.-A. (2010). The impact of body mass index on the prevalence of low back pain. *Spine*.

[36] Charles A. S., David J. K., Samuel L. Q., Ahn M. U., Nicholas U. A. (2018). The association between body mass index and the prevalence, severity, and frequency of low back pain: data from the osteoarthritis initiative. *Spine*.

[37] Hales T. R., Bernard B. P. (1996). Epidemiology of work-related musculoskeletal disorders. *Orthopedic Clinics of North America*.

[38] Bongers P. M., de Winter C. R., Kompier M. A., Hildebrandt V. H. (1993). Psychosocial factors at work and musculoskeletal disease. *Scandinavian Journal of Work, Environment and Health*.

[39] Erick P. N., Smith D. R. (2014). Low back pain among school teachers in Botswana, prevalence and risk factors. *BMC Musculoskeletal Disorders*.

[40] Zhao I., Bogossian F., Turner C. (2010). Shift work and work related injuries among health care workers: a systematic review. *Australian Journal of Advanced Nursing*.

[41] Amer S. A. (2018). Work-related musculoskeletal symptoms among nurse staff in Ismailia, Egypt. Egypt. *Journal of Occupational Medicine*.

[42] Sikiru L., Hanifa S. (2010). Prevalence and risk factors of low back pain among nurses in a typical Nigerian hospital. *African Health Sciences*.

[43] Bin Homaid M., Abdelmoety D., Alshareef W. (2016). Prevalence and risk factors of low back pain among operation room staff at a Tertiary Care Center, Makkah, Saudi Arabia: a cross-sectional study. *Annals of Occupational and Environmental Medicine*.

[44] Samat R. A., Shafei M. N., Yaacob N. A., Yusoff A. (2011). Prevalence and associated factors of back pain among dental personnel in North-Eastern State of Malaysia. *International Journal of Collaborative Research on Internal Medicine and Public Health*.

[45] Burdorf A., Sorock G. (1997). Positive and negative evidence of risk factors for back disorders. *Scandinavian Journal of Work, Environment and Health*.

[46] Hoogendoorn W. E., van Poppel M. N. M., Bongers P. M., Koes B. W., Bouter L. M. (2000). Systematic review of psychosocial factors at work and private life as risk factors for back pain. *Spine*.

[47] Luchini C., Stubbs B., Solmi M., Veronese N. (2017). Assessing the quality of studies in meta-analyses: advantages and limitations of the newcastle ottawa scale. *World Journal of Meta-Analysis*.

[48] JBI (2017). *The Joanna Briggs Institute Critical Appraisal Tools for Use in JBI Systematic Reviews: Checklist for Case Control Studies*.

[49] Huedo-Medina T. B., Sánchez-Meca J., Marín-Martínez F., Botella J. (2006). Assessing heterogeneity in meta-analysis: *Q* statistic or *I*² index?. *Psychological Methods*.

[50] DerSimonian R., Laird N. (1986). Meta-analysis in clinical trials. *Controlled Clinical Trials*.

[51] Egger M., Davey-Smith G., Altman D. (2008). *Systematic Reviews in Health Care: Meta-Analysis in Context*.

[52] Flegal K. M., Kit B. K., Graubard B. I. (2014). Body mass index categories in observational studies of weight and risk of death. *American Journal of Epidemiology*.

[53] Macdonald S., Maclntyre P. (1997). The generic job satisfaction scale. *Employee Assistance Quarterly*.

[54] Rolander B., Bellner A. L. (2001). Experience of musculo-skeletal disorders, intensity of pain, and general conditions in work -- the case of employees in non-private dental clinics in a county in southern Sweden. *Work*.

[55] Shukla A., Srivastava R. (2016). Development of short questionnaire to measure an extended set of role expectation conflict, coworker support and work-life balance: the new job stress scale. *Cogent Business and Management*.

[56] Nakata A., Ikeda T., Takahashi M. (2006). The prevalence and correlates of occupational injuries in small‐scale manufacturing enterprises. *Journal of Occupational Health*.

[57] Wami S. D., Abere G., Dessie A., Getachew D. (2019). Work-related risk factors and the prevalence of low back pain among low wage workers: results from a cross-sectional study. *BMC Public Health*.

[58] Mekonnen T. H. (2019). Work-related factors associated with low back pain among nurse professionals in east and west wollega zones, western Ethiopia, 2017: a cross-sectional study. *Pain and Therapy*.

[59] Mekonnen T. H. (2019). The magnitude and factors associated with work-related back and lower extremity musculoskeletal disorders among barbers in Gondar town, northwest Ethiopia, 2017: a cross-sectional study. *PLoS One*.

[60] Deksisa Abebe A., Gebrehiwot E. M., Lema S., Abebe T. W. (2015). Prevalence of low back pain and associated risk factors among Adama Hospital Medical College Staff, Ethiopia. *European Journal of Preventive Medicine*.

[61] Abebaw T.-A., Weldegebriel M. K., Gebremichael B., Abaerei A. A. (2018). Prevalence and associated factors of low back pain among teachers working at governmental primary schools in Addis Ababa, Ethiopia: a cross sectional study. *Biomedical Journal*.

[62] Yosef T., Belachew A., Tefera Y. (2019). Magnitude and contributing factors of low back pain among long distance truck drivers at Modjo dry port, Ethiopia: a cross-sectional study. *Journal of environmental and public health*.

[63] Kebede A., Abebe S. M., Woldie H., Yenit M. K. (2019). Low back pain and associated factors among primary school teachers in mekele city, north Ethiopia: a cross-sectional study. *Occupational Therapy International*.

[64] Beyen T. K., Mengestu M. Y., Zele Y. T. (2013). Low back pain and associated factors among teachers in gondar town, north gondar, Amhara region, Ethiopia. *Occupational Medicine and Health Affairs*.

[65] Belay M. M., Worku A., Gebrie S., Wamisho B. (2016). Epidemiology of low back pain among nurses working in public hospitals of Addis Ababa, Ethiopia. *East and Central African Journal of Surgery*.

[66] Wanamo M. E., Abaya S. W., Aschalew A. B. (2017). Prevalence and risk factors for low back pain (LBP) among Taxi Drivers in Addis Ababa, Ethiopia: a community based cross-sectional study. *The Ethiopian Journal of Health Development*.

[67] Assefa H. (2017). *Prevalence and Risk Factors of Low Back Pain in Nurses Working at Tikur Anbessa Specialized Hospital and Zewditu Memorial Hospital, Addis Ababa, Ethiopia*.

[68] Tefera Y., Ahmed A. N., Wondie Y. (2019). Prevalence of low back pain and associated factors among young workers in traditional weaving of the informal sectors, Central and Southern Ethiopia. *Vulnerable Children and Youth Studies*.

[69] Tafese A. (2018). Occupational risk factors of low back pain among ammunition engineering industry in West Shoa Zone, Ethiopia, 2017. *Journal of Medicine, Physiology and Biophysics*.

[70] Tafese A., Kebede G., Shibiru A., Benti T. (2018). Work-related low back pain among garment industry workers in Eastern Oromia Region, Ethiopia. *International Journal of Occupational Hygiene*.

[71] Assefa T. (2017). *Prevalnce of Work Related Lower Back Pain and Assocaiated Factors Among Welders in Selected Metal and Engineering Industries in Addis Ababa and Surrounding Towns*.

[72] Yitayeh A., Mekonnen S., Fasika S., Gizachew M. (2015). Annual prevalence of self-reported work related musculoskeletal disorders and associated factors among nurses working at Gondar Town Governmental Health Institutions, Northwest Ethiopia. *Emergency Medicine*.

[73] Lamina S., Shmaila H., Amaeze A. A., Subramania M.-B. (2009). Prevalence and risk factors of low back pain among nurses in Africa: Nigerian and Ethiopian specialized hospitals survey study. *East African Journal of Public Health*.

[74] Henok A., Bekele T. (2017). Prevalence of musculoskeletal pain and factors associated with kyphosis among pedestrian back-loading women in selected towns of Bench Maji zone, Southwest Ethiopia. *The Ethiopian Journal of Health Development*.

[75] Lette A., Hussen A., Kumbi M., Nuriye S., Lamore Y. (2019). Musculoskeletal pain and associated factors among building construction workers in southeastern Ethiopia. *International Journal of Industrial Ergonomics*.

[76] Girma Z. (2016). *Assessing the Prevalence of Work Related Musculoskeletal Disorders and Associated Factors Among Workers in Selected Garments in Addis Ababa, Ethiopia*.

[77] Bedru W. (2016). *Self-Reported Work Related Musculoskeletal Disorders and Determinant Factors of Female Beauty Salon Hair Dressers*.

[78] Etana G. (2019). *Prevalence of Work Related Musculoskeletal Disorder and Associated Factors Among Bank Staff in Jimma City, Southwest Ethiopia*.

[79] Regassa T. M., Lema T. B., Garmomsa G. N. (2018). Work related musculoskeletal disorders and associated factors among nurses working in Jimma Zone Public Hospitals, South West Ethiopia. *Occupational Medicine and Health Affairs*.

[80] Hailu W., Getahun M., Mohammed A. (2018). *Assessment of Back Pain and Disability Status Among Automotive Industry Workers, Ethiopia*.

[81] Zungu L. I., Nigatu E. S. (2015). A comparative study of the prevalence and risk factors of lower back pain among aircraft technicians in Ethiopian Airlines. *Occupational Health South Africa*.

[82] Deksisa A., Garoma S., Dugasa W. (2019). Factors associated with lower back pain among adult patients at selected health institutions in Adama Town, Ethiopia. *International Journal of Advanced Research and Publications*.

[83] Yehualaw W. (2017). *Assessment of Self-Reported Work Related Low Back Pain and Associated Factors Among Nurses Working in Intensive Care Unit (ICU) at Public and Private Hospitals, Addis Ababa, Ethiopia*.

[84] Teklu S. (2017). *Assessment of Prevalence and Associated Factors of Work Related Musculoskeletal Disorders Among Cobble Stone Workers in Addis Ababa, Ethiopia*.

[85] Fekadu Mijena G., Geda B., Dheresa M., Fage S. G. (2020). Low back pain among nurses working at public hospitals in eastern Ethiopia. *Journal of Pain Research*.

[86] Abir T., Wogderess S., Yigremew T. (2017). Assessing the existence of work related musculoskeletal disorders and their influences on office workers in some selected woredas of North Showa Zone. *Journal of Physical Education, Sports Management and Yogic Sciences*.

[87] Jeon M.-j., Jeon H., Jeon H.-s. (2017). Musculoskeletal pain status of local farmers in Tigray, Ethiopia: a cross-sectional survey. *Physical Therapy Korea*.

[88] Delele M., Janakiraman B., Bekele Abebe A., Tafese A., van de Water A. T. M. (2018). Musculoskeletal pain and associated factors among Ethiopian elementary school children. *BMC Musculoskeletal Disorders*.

[89] Bello B., Adebayo H. B. (2017). A systematic review on the prevalence of low back pain in Nigeria. *Middle East Journal of Rehabilitation and Health Studies*.

[90] Dionne C. E., Dunn K. M., Croft P. R. (2006). Does back pain prevalence really decrease with increasing age? A systematic review. *Age and Ageing*.

[91] De Souza I. M. B., Sakaguchi T. F., Yuan S. L. K. (2019). Prevalence of low back pain in the elderly population: a systematic review. *Clinics*.

[92] Jeffries L. J., Milanese S. F., Grimmer-Somers K. A. (2007). Epidemiology of adolescent spinal pain: a systematic overview of the research literature. *Spine (Phila Pa 1976)*.

[93] Calvo-Muñoz I., Gómez-Conesa A., Sánchez-Meca J. (2013). Prevalence of low back pain in children and adolescents: a meta-analysis. *BMC Pediatrics*.

[94] Kopec J. A., Sayre E. C., Esdaile J. M. (2004). Predictors of back pain in a general population cohort. *Spine (Phila Pa 1976)*.

[95] Waxman R., Tennant A., Helliwell P. (2000). A prospective follow-up study of low back pain in the community. *Spine*.

[96] Emmanuel M. N., Ezhilarasu P., Bheemarao A. B. (2015). Low back pain among nurses in a Tertiary Hospital, South India. *Journal of Osteoporosis and Physical Activity*.

[97] Barkhordari A., Halvani G., Barkhordari M. (2013). The prevalence of low back pain among nurses in Yazd, Southeast Iran. *International Journal of Occupational Hygiene*.

[98] Manandhar N., Subedi S. (2016). Prevalence and risk factors of low back pain among nurses of a Medical College at Bharatpur, Nepal. *SCIREA Journal of Health*.

[99] Mutanda T., Mwaka E., Sekimpi P., Juliet Ntuulo M. (2017). Occupation related musculoskeletal disorders among nurses at the National Referral Hospital, Mulago in Uganda. *Occupational Medicine and Health Affairs*.

[100] Semachew A., Workineh Y., Ayalew E., Animaw W. (2018). Low back pain among nurses working in a clinical settings of Africa: a systematic review and meta-analysis of a 19 years of studies. *BioRxiv*.

[101] Ahmadi H., Farshad A. A., Motamed Z. M., Mahjoub H. (2014). Epidemiology of low-back pain and its association with occupational and personal factors among employees of hamadan province industries. *Journal of Health and Hygiene*.

[102] Murtezani A., Ibraimi Z., Sllamniku S., Osmani T., Sherifi S. (2011). Prevalence and risk factors for low back pain in industrial workers. *Prevalence*.

[103] Sanya A. O., Ogwumike O. O. (2005). Low back pain prevalence amongst industrial workers in the private sector in Oyo State, Nigeria. *African Journal of Medicine and Medical Sciences*.

[104] Boden S. D., Davis D. O., Dina T. S., Patronas N. J., Wiesel S. W. (1990). Abnormal magnetic-resonance scans of the lumbar spine in asymptomatic subjects. A prospective investigation. *Journal of Bone and Joint Surgery*.

[105] Guo H.-R. (2002). Working hours spent on repeated activities and prevalence of back pain. *Occupational and Environmental Medicine*.

[106] Yip Y. b. (2001). A study of work stress, patient handling activities and the risk of low back pain among nurses in Hong Kong. *Journal of Advanced Nursing*.

[107] Troup J. D. (1984). Causes, prediction and prevention of back pain at work. *Scandinavian Journal of Work, Environment and Health*.

[108] Brown J. R. (1975). Factors contributing to the development of low back pain in industrial workers. *American Industrial Hygiene Association Journal*.

[109] Magora A. (1975). Investigation of the relation between low back pain and occupation. VII. Neurologic and orthopedic condition. *Scandinavian Journal of Rehabilitation Medicine*.

[110] Riihimäki H. (1991). Low-back pain, its origin and risk indicators. *Scandinavian Journal of Work, Environment and Health*.

[111] Luoma K., Riihimäki H., Raininko R., Luukkonen R., Lamminen A., Viikari-Juntura E. (1998). Lumbar disc degeneration in relation to occupation. *Scandinavian Journal of Work, Environment & Health*.

[112] Erick P. N., Smith D. R. (2011). A systematic review of musculoskeletal disorders among school teachers. *BMC Musculoskeletal Disorders*.

[113] Chaffin D. B., Park K. S. (1973). A longitudinal study of low-back pain as associated with occupational weight lifting factors. *American Industrial Hygiene Association Journal*.

[114] Amiri S., Behnezhad S. (2020). Sleep disturbances and back pain: systematic review and meta-analysis. *Neuropsychiatrie*.

[115] Hincapié C. A., Cassidy J. D., Côté P. (2008). Is a history of work-related low back injury associated with prevalent low back pain and depression in the general population?. *BMC Musculoskeletal Disorders*.

[116] Luan H. D., Hai N. T., Xanh P. T. (2018). Musculoskeletal disorders: prevalence and associated factors among district hospital nurses in Haiphong, Vietnam. *BioMed Research International*.

[117] Griffin D. W., Harmon D. C., Kennedy N. M. (2012). Do patients with chronic low back pain have an altered level and/or pattern of physical activity compared to healthy individuals? A systematic review of the literature. *Physiotherapy*.

[118] Sitthipornvorakul E., Janwantanakul P., Purepong N., Pensri P., van der Beek A. J. (2011). The association between physical activity and neck and low back pain: a systematic review. *European Spine Journal*.

[119] Hosam A., Mackey M., Stamatakis E., Zadro J. R., Shirley D. (2019). The association between physical activity and low back pain: a systematic review and meta-analysis of observational studies. *Scientific Reports*.

[120] Heneweer H., Staes F., Aufdemkampe G., van Rijn M., Vanhees L. (2011). Physical activity and low back pain: a systematic review of recent literature. *European Spine Journal*.

