# Supplementary material for: Prevalence of Self-Reported Work-Related Lower Back Pain and Its Associated Factors in Ethiopia: A Systematic Review and Meta-Analysis
Source: J Environ Public Health. 2021 Sep 23;2021:6633271. doi: 10.1155/2021/6633271 (PMC8486508; doi:10.1155/2021/6633271)
Supplement: Supplementary Materials — The methodological quality of the included studies was assessed using “Newcastle-Ottawa Quality Assessment Tool Adapted for Cross-Sectional Studies” shown in Additional file 1. The search strategy and results obtained using PubMed are shown in Additional file 2. [file 6633271.f1.zip › 6633271.f1/Additional file 1.docx]

Table 1: Methodological appraisal tool: Newcastle-Ottawa Quality Assessment tool adapted for cross-sectional studies

|  | **Criteria** | **Yes/No** |
| --- | --- | --- |
| **A** | **Is the final sample representative of the target population?** |  |
| **1** | At least one of the following must apply in the study: an entire target population, randomly selected sample or sample stated to represent the target population. |  |
| **2** | At least one of the following: reasons for non-response described, non-responders described, comparison of responders and non-responders, or comparison of sample and target population. |  |
| **3** | Response rate, and if applicable, drop-out rate reported |  |
| **B** | **Quality of data** |  |
| **4** | Were the data primary data of LBP, or was it taken from a survey not specifically designed for that purpose? |  |
| **5** | Were the data collected from each subject directly or were they collected from a proxy? |  |
| **6** | Was the same mode of data collection used for all subjects? |  |
| **7** | At least 1 of the following in case of:  a) Questionnaire: a validated questionnaire or at least tested for reproducibility?  b) Interview: interview validated, tested for reproducibility, or adequately described and standardized?  c) Examination: examination validated, tested for reproducibility, adequately described and standardized? |  |
| **C** | **Definition of LBP** |  |
| **8** | Was there a precise anatomic delineation of the lumbar area or reference to an easily obtainable article that contains such specification? |  |
| **9** | Was there further useful specification of the definition of LBP, or question(s) put to study subjects quoted such as frequency, duration, or intensity, and character of the pain. Or was there reference to an easily obtainable article that contains such specification? |  |
| **10** | Were the recall periods clearly stated: e.g. 1 week, 1 month, lifetime? |  |
|  | **Total score** | **100** |

**Table 2: Methodological appraisal tool: JBI Critical Appraisal Checklist for Case Control Studies**

**Reviewer Date**

**Author Year Record Number**

| **Criteria** | **Yes** | **No** | **Unclear** | **Not applicable** |
| --- | --- | --- | --- | --- |
| 1. Were the groups comparable other than the presence of disease in cases or the absence of disease in controls? | □ | □ | □ | □ |
| 1. Were cases and controls matched appropriately? | □ | □ | □ | □ |
| 1. Were the same criteria used for identification of cases and controls? | □ | □ | □ | □ |
| 1. Was exposure measured in a standard, valid and reliable way? | □ | □ | □ | □ |
| 1. Was exposure measured in the same way for cases and controls? | □ | □ | □ | □ |
| 1. Were confounding factors identified? | □ | □ | □ | □ |
| 1. Were strategies to deal with confounding factors stated? | □ | □ | □ | □ |
| 1. Were outcomes assessed in a standard, valid and reliable way for cases and controls? | □ | □ | □ | □ |
| 1. Was the exposure period of interest long enough to be meaningful? | □ | □ | □ | □ |
| 1. Was appropriate statistical analysis used? | □ | □ | □ | □ |

Overall appraisal: Include □ Exclude □ Seek further info □

Comments (Including reason for exclusion)
