# Supplementary material for: Prevalence of Self-Reported Work-Related Lower Back Pain and Its Associated Factors in Ethiopia: A Systematic Review and Meta-Analysis
Source: J Environ Public Health. 2021 Sep 23;2021:6633271. doi: 10.1155/2021/6633271 (PMC8486508; doi:10.1155/2021/6633271)
Supplement: Supplementary Materials — The methodological quality of the included studies was assessed using “Newcastle-Ottawa Quality Assessment Tool Adapted for Cross-Sectional Studies” shown in Additional file 1. The search strategy and results obtained using PubMed are shown in Additional file 2. [file 6633271.f1.zip › 6633271.f1/Additional File 2.docx]

|  |  | **PubMed Search Strategy** |  |  |
| --- | --- | --- | --- | --- |
| [#1](https://www.ncbi.nlm.nih.gov/pubmed) | [Add](https://www.ncbi.nlm.nih.gov/pubmed) | Search (((Prevalence) OR Magnitude) OR Epidemiology) OR Burden | [3002677](https://www.ncbi.nlm.nih.gov/pubmed/?cmd=HistorySearch&querykey=1) | 11:06:21 |
| [#2](https://www.ncbi.nlm.nih.gov/pubmed) | [Add](https://www.ncbi.nlm.nih.gov/pubmed) | Search (((Associated factors [Title/Abstract]) OR risk factors [Title/Abstract]) OR Work-related factors [Title/Abstract]) OR Predictors [Title/Abstract] | [632452](https://www.ncbi.nlm.nih.gov/pubmed/?cmd=HistorySearch&querykey=2) | 11:09:36 |
| [#3](https://www.ncbi.nlm.nih.gov/pubmed) | [Add](https://www.ncbi.nlm.nih.gov/pubmed) | Search (((Low back pain) AND Back pain) AND Musculoskeletal disorder) AND Musculoskeletal Pain Sort by: Best Match | [1906](https://www.ncbi.nlm.nih.gov/pubmed/?cmd=HistorySearch&querykey=3) | 11:10:57 |
| [#4](https://www.ncbi.nlm.nih.gov/pubmed) | [Add](https://www.ncbi.nlm.nih.gov/pubmed) | Search (((Low* back pain[Title/Abstract]) OR Back pain[Title/Abstract]) OR Musculoskeletal disorder[Title/Abstract]) OR Musculoskeletal Pain[Title/Abstract] | [50851](https://www.ncbi.nlm.nih.gov/pubmed/?cmd=HistorySearch&querykey=4) | 11:14:05 |
| [#5](https://www.ncbi.nlm.nih.gov/pubmed) | [Add](https://www.ncbi.nlm.nih.gov/pubmed) | Search "Ethiopia"[Title/Abstract] | [13963](https://www.ncbi.nlm.nih.gov/pubmed/?cmd=HistorySearch&querykey=5) | 11:16:50 |
| [#6](https://www.ncbi.nlm.nih.gov/pubmed) | [Add](https://www.ncbi.nlm.nih.gov/pubmed) | Search (((((((Prevalence) OR Magnitude) OR Epidemiology) OR Burden)) AND ((((Associated factors[Title/Abstract]) OR risk factors[Title/Abstract]) OR Work-related factors [Title/Abstract]) OR Predictors[Title/Abstract])) AND ((((Low* back pain[Title/Abstract]) OR Back pain [Title/Abstract]) OR Musculoskeletal disorder[Title/Abstract]) OR Musculoskeletal Pain [Title/Abstract])) AND "Ethiopia"[Title/Abstract] | [9](https://www.ncbi.nlm.nih.gov/pubmed/?cmd=HistorySearch&querykey=7) | 11:23:07 |
